# Editorial: The good side of technology: how we can harness the positive potential of digital technology to maximize well-being

**DOI:** 10.3389/fpsyg.2023.1304592

**Published:** 2023-10-11

**Authors:** John F. Hunter, Lisa C. Walsh, Chi-Keung Chan, Stephen M. Schueller

**Affiliations:** ^1^Department of Psychology, Chapman University, Orange, CA, United States; ^2^Department of Psychology, University of California, Los Angeles, Los Angeles, CA, United States; ^3^School of Arts and Humanities, Tung Wah College, Hong Kong, Hong Kong SAR, China; ^4^Department of Psychological Science, University of California, Irvine, Irvine, CA, United States

**Keywords:** digital technology, positive psychology, wellbeing, mental health, cyberpsychology, smartphones, social media, artificial intelligence

The rapid advancement of digital technology has transformed society and undeniably impacted wellbeing. With the advent of smartphones and social media, a host of empirical articles, popular press pieces, non-fiction books, and documentaries have highlighted the potential negative effects of technology, such as addiction, loneliness, and depression (e.g., Alter, 2017; Twenge et al., 2018; Orlowski, 2020). While it is important to acknowledge and address the potentially detrimental effects of this increasing technological reliance, we believe that it is imperative that researchers, developers, and users embrace a more balanced approach that also recognizes the positive potential of digital technology to support wellbeing. This Research Topic demonstrates a variety of ways in which technological tools can be both designed and used to maximize wellbeing across a range of domains. These studies collectively emphasize a critical message: for the most part technology itself is neither good nor bad, but how technological affordances are harnessed determines their impact on wellbeing. While we cannot ignore potential pitfalls, recognizing and leveraging the positive potential of digital technology is a paramount endeavor as technology becomes further integrated in our lives.

Technological affordances (i.e., the potential actions technology affords to its human users), particularly in the realm of digital technology, have evolved considerably in the past few decades (Conole and Dyke, 2004; Parchoma, 2014). Leveraging these affordances appropriately may allow us to foster greater wellbeing and more social connection in various contexts. This approach is aligned with the perspectives of the positive technology movement that seeks to draw on technology and wellbeing science to optimize psychological and physical health (Riva et al., 2012; Gaggioli et al., 2019). The articles in this issue advance this approach by highlighting several areas in which technology may be a force for good. Rosič et al. unveiled the positive facets of youth digital interactions through the Digital Flourishing Scale, emphasizing areas like connectedness and authentic self-presentation. This counters the narrative that technology always inherently harms youth mental health. Lee et al. introduced the concept of agentic social media use, establishing the importance of intentional, meaningful engagement. During the pandemic's enforced isolation, Heyman and Kushlev showed how smartphones ensured sustained connections and information access. Similarly, Petersen et al. emphasized how technology may be used to reduce loneliness, especially in older adults, who are more prone to isolation. Similarly, Chase et al. revealed that digital technology, when used for social ties, has minimal negative ramifications and can actually bolster mental health among emerging adults. Each of these contributions provide evidence to suggest that purposeful socially-motivated online behavior is positively associated with indicators of wellbeing.

Many of the studies in this issue also demonstrate the potential of digital technology to reach traditionally underserved and marginalized populations (Schueller et al., 2019) by illustrating how these tools are particularly well-suited for tailoring culturally-sensitive interventions that can impact populations across the lifespan and around the world. Liu et al. explored the positive outcomes of smartwatch use on user reciprocity through expanding users' social relationships and increasing their social engagement in mainland China. Marciano and Viswanath provided evidence that certain social media activities can enhance Swiss adolescents' flourishing by fulfilling their basic needs. Zhong et al. showed that internet use can be beneficial for Chinese residents if used appropriately. Wu et al. study also echoed the positive use of social media when coupled with strong digital skills on the wellbeing of Chinese residents. Shi and Khoo demonstrated the positive changes in self-disclosure and social networks through an online health community for Chinese users with depressive symptoms. These studies exhibit how digital technology is a universally impactful factor around the globe, especially in regard to how it can address the need for belongingness by building avenues that supplement social interaction. The continued integration of cross-cultural perspectives in the development and use of technology will enrich our understanding of the positive potential of these tools.

One challenge with understanding the positive potential of technologies is that doing so requires expertise and content knowledge that draws from diverse fields—psychology and wellbeing science, technology and human-computer interaction, evaluation, and clinical methodologies—and coordination across industry, academia, policymakers, and other invested parties. The papers in this Research Topic emphasize this need for work that engages interdisciplinarily and thus create frameworks and models to advance this space. For example, Villamil and Heshmati propose an Engagement in the Good with Technology (EGT) Framework with implications for digital technology research and design. One research implication of the EGT Framework is to consider not just positive or negative use, but to understand the ratio of positive to negative interactions with technology. Such an approach highlights that neither positive nor negative use take place in isolation and each individual has to balance the potential benefits and risks. Also, when research measures both positives and negatives, it provides the opportunity for researchers to understand the relative impact on each. From a design perspective, EGT provides an additional design target. In their development, the functionality of technologies is considered, not their impact. Elevating “good” as a design feature may lead to the development of features that can function optimally with positive impact.

This interdisciplinary perspective is especially important to consider in light of the evolving regulatory discussions around technologies. The apparent harms of social media and possible risks of artificial intelligence implementation present challenges for policy and regulation. Technologies are constantly changing and regulatory perspectives, focused on using punishments (or “sticks”) to shape technologies, will likely always lag behind technology development and fail to motivate companies to develop better products. A positive framework for technology development could identify ways to incentivize companies (using “carrots”) and provide actionable insights to team with companies to create wellbeing promoting products. Aligned with this thinking, we need design frameworks that specify how to design for wellbeing, like Liedgren et al. liminal design in this Research Topic, and evaluation frameworks that help demonstrate the success of the design.

Industry teams are traditionally not siloed by discipline in the same way that academic teams are divided by traditional disciplines like psychology, computer science, and public health. Interdisciplinary journals, like this one, conferences, and projects are beginning to create spaces to engage in this dialogue. One major challenge, however, in deeper interdisciplinary research is ensuring that research teams from different disciplines can engage on a research level. That is ensuring that technologists are not merely included to “build the stuff” that psychologists want to use to deliver their experiences or evaluate and that psychologists are not merely included to identify clinical areas of need or evidence-based interventions. A truly collaborative and transdisciplinary approach will be necessary to ensure that the future of technological development is undertaken ethically, efficiently, and effectively.

The articles in this Research Topic not only enrich our intellectual and practical knowledge about the good side of digital technology to support mental health, but also serve as a call to others to shift their perspective and embrace the responsibility of working together to design and use technology to maximize wellbeing. We are not advocating that everyone blindly support the inevitable immersion of technology ever deeper into our lives with optimistic ignorance. Rather, we urge others to acknowledge the permanence of digital technology as a mainstay in our future so that we can work collaboratively and constructively to harness its positive potential for the advancement of human wellness. In the coming years, artificial intelligence, immersive interfaces (e.g., virtual and augmented reality), and a series of yet-to-be imagined technologies will continue to reshape the dynamics of human interaction and wellbeing. As researchers designing and studying these tools, and as users relying on and interacting with these technologies, we are faced with a critical decision about how we proceed. Do we fight against the inevitable tide of development with doomsday predictions and laments of misguided trends? Or do we collectively embrace the gifts of the digital age to foster social relationships and build connective bridges, address inequities and inefficiencies in our industries and societies, and develop new paths forward that will allow us to thrive and flourish with technology in hand?

## Author contributions

JH: Writing—original draft, Writing—review and editing. LW: Writing—original draft, Writing—review and editing. C-KC: Writing—original draft, Writing—review and editing. SS: Writing—original draft, Writing—review and editing.

## References

[B1] Alter A. (2017). Irresistible: The Rise of Addictive Technology and the Business of Keeping us Hooked. New York, Penguin press.

[B2] Conole G. Dyke M. (2004). What are the affordances of information and communication technologies? ALT-J. Res. Learn. Technol. 12, 113–124. 10.3402/rlt.v12i2.11246

[B3] Gaggioli A. Villani D. Serino S. Baños R. M. Botella C. (2019). Positive technology designing e-experiences for positive change. Front. Psychol. 10, 1571. 10.3389/fpsyg.2019.01571 31338053 PMC6629820

[B4] Orlowski J. (2020). The Social Dilemma L. Rhodes. Boulder, CO: Netflix.

[B5] Parchoma G. (2014). The contested ontology of affordances: Implications for researching technological affordances for collaborative knowledge production. Comput. Hum. Behav. 37, 360–368. 10.1016/j.chb.2012.05.028

[B6] Riva G. Baños R. M. Botella C. Wiederhold B. K. Gaggioli A. (2012). Positive technology: Using interactive technologies to promote positive functioning. Cyberpsychol. Behav. Soc. Netw. 15, 69–77. 10.1089/cyber.2011.0139 22149077

[B7] Schueller S. M. Hunter J. F. Figueroa C. Aguilera A. (2019). Use of digital mental health for marginalized and underserved populations. Curr. Treatm. Opti. Psychiat. 6, 243–255. 10.1007/s40501-019-00181-z

[B8] Twenge J. M. Joiner T. E. Rogers M. L. Martin G. N. (2018). Increases in depressive symptoms, suicide-related outcomes, and suicide rates among U.S. Adolescents after 2010 and links to increased new media screen time. Clin. Psychol. Sci. 6, 3–17. 10.1177/2167702617723376

